# Reply: The forced correlation between ISCHEMIA and the inaccurate CABG recommendations of the 2021 American College of Cardiology/American Heart Association/Society for cardiovascular Angiography coronary revascularization guidelines

**DOI:** 10.1016/j.xjon.2022.04.024

**Published:** 2022-04-21

**Authors:** Faisal G. Bakaeen, Marc Ruel, Leonard N. Girardi, Joseph F. Sabik

**Affiliations:** a Coronary Center, Department of Thoracic and Cardiovascular Surgery, Heart, Vascular and Thoracic Institute Cleveland Clinic, Cleveland, Ohio; b Division of Cardiac Surgery, University of Ottawa, Ottawa, Ontario, Canada; c NewYork-Presbyterian/Weill Cornell Medical Center, New York, NY; d Department of Surgery, University Hospitals Cleveland Medical Center, Case Western Reserve University, Cleveland, Ohio

Reply to the Editor:

**Figure:**
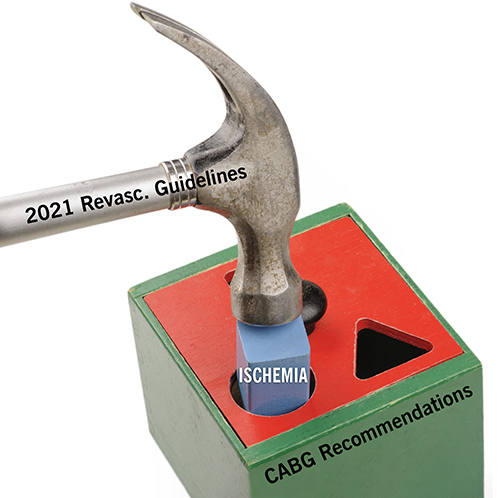

The authors reported no conflicts of interest.

The *Journal* policy requires editors and reviewers to disclose conflicts of interest and to decline handling or reviewing manuscripts for which they may have a conflict of interest. The editors and reviewers of this article have no conflicts of interest.

We appreciate the Letter to the Editor by Maron and colleagues regarding our manuscript addressing reasons for not endorsing the 2021 American College of Cardiology/American Heart Association/Society for Cardiovascular Angiography and Interventions Coronary Revascularization Guidelines.^1^, ^2^, ^3^ First, we congratulate the authors of the ISCHEMIA trial (International Study of Comparative Health Effectiveness With Medical and Invasive Approaches) report on a well-conducted study providing reassuring data regarding the safety of initial conservative management of patients with low atherosclerotic burden and normal ventricular function. However, we strongly believe that ISCHEMIA offers no meaningful data to inform coronary artery bypass grafting (CABG) recommendations for multivessel coronary artery disease (CAD) in the contested guidelines,^3^ nor any data to invalidate the previous Class 1 Level of Evidence A for CABG in multivessel disease.^4^ Nevertheless, ISCHEMIA provided the “new evidence” cited by the American College of Cardiology/American Heart Association/Society for Cardiovascular Angiography and Interventions Coronary Revascularization Guidelines Writing Committee to downgrade CABG to class 2b for survival benefit in patients with multivessel disease and normal ejection fraction.^3^

The typical multivessel CABG referral by modern-day heart teams is for severe (≥70% stenosis) 3-vessel CAD or severe 2-vessel disease including a proximal left anterior descending (LAD) lesion (ie, modified Duke Prognostic Index score of 6, as defined by the authors). Only 40 patients were in the latter category in the ISCHEMIA trial, according to Tables E1 and E2, “Baseline Characteristics by Ischemia Severity” and “Baseline Characteristics by Ischemia Severity and Treatment group,” respectively.^5^ If these tables are accurate, then the authors may have inadvertently upgraded all the Duke score assignments related to CAD extent and severity in subsequent data tables, an error perpetuated in the data analysis and corresponding manuscript.^5^ Thus, no actual Duke 6 data were presented, and Duke 5 (2-vessel severe stenosis not including the proximal LAD, 1-vessel severe proximal LAD, or 3-vessel moderate stenosis [≥50%], N = 659) data were presented instead. Despite that, a significant reduction occurred in the 4-year rate of cardiovascular death or myocardial infarction in the invasive strategy group (difference, 6.3% [95% confidence interval, 0.2%-12.4%]).^5^ Thus, invasive strategy was associated with better event-free survival and a decreasing trend in cardiovascular mortality even in patients with less severe CAD than those referred routinely for CABG.^5^ In addition, we had previously discussed why CABG and percutaneous coronary intervention are different treatment modalities with different indications and outcomes and should not be lumped together as equivalent revascularization procedures.^2^

Finally, more of the patients in the invasive-strategy group of ISCHEMIA received medical therapy only than CABG. As such, a comparison of the invasive-strategy group to the conservative-strategy—or “medical therapy”—group does not evaluate the effects of CABG. Furthermore, representative controls to the proportionally few patients who received CABG—plausibly those who presented with the most angiographically severe CAD—cannot be obtained from the conservative-strategy group, as patients in the latter group did not undergo coronary angiography.

In view of the aforementioned, we ask that Maron and colleagues provide clarifications and, if our concerns are confirmed, that appropriate action be taken, including correcting the current Letter to the Editor^1^ and all affected ISCHEMIA publications.^1^ We maintain that ISCHEMIA is a useful study, but unfortunately, its scope and findings were stretched beyond their original bounds. We strongly believe that unchecked interpretations of ISCHEMIA could lead to inaccurate recommendations, depriving countless patients of the longevity associated with CABG in severe multivessel disease.
